# Extra-Virgin Olive Oil from Apulian Cultivars and Intestinal Inflammation

**DOI:** 10.3390/nu12041084

**Published:** 2020-04-14

**Authors:** Marica Cariello, Annalisa Contursi, Raffaella Maria Gadaleta, Elena Piccinin, Stefania De Santis, Marilidia Piglionica, Ada Fiorenza Spaziante, Carlo Sabbà, Gaetano Villani, Antonio Moschetta

**Affiliations:** 1 Clinica Medica Cesare Frugoni, Department of Interdisciplinary Medicine, University of Bari “Aldo Moro”, 70124 Bari, Italy; maricacariello@gmail.com (M.C.); r.gadaleta80@gmail.com (R.M.G.); elena.piccinin@gmail.com (E.P.); marilidia.piglionica1@gmail.com (M.P.); carlo.sabba@uniba.it (C.S.); 2 Department of Neuroscience, Imaging, and Clinical Sciences and Center for Research on Aging and Translational Medicine, “G. d’Annunzio” University School of Medicine, 66100 Chieti, Italy; annalisa.contursi@virgilio.it; 3 Department of Pharmacy-Drug Science, University of Bari “Aldo Moro”, 70126 Bari, Italy; stefania.desantis@uniba.it; 4 Department of Basic Medical Sciences, Neurosciences and Sense Organs, University of Bari “Aldo Moro”, 70124 Bari, Italy; ada.spaziante@virgilio.it (A.F.S.); gaetano.villani@uniba.it (G.V.); 5 INBB, National Institute for Biostructures and Biosystems, 00136 Rome, Italy; 6 IRCCS Istituto Tumori Giovanni Paolo II, 70124 Bari, Italy

**Keywords:** olive oil, inflammatory bowel disease, intestinal inflammation, animal experimentation, mouse model

## Abstract

Inflammatory bowel disease (IBD) is a multifactorial intestinal disorder characterized by chronic intestinal inflammation. The etiology of IBD is still unclear, although genetic, environmental and host factors have been associated to the disease. Extra-virgin olive oil (EVO) is a central component of the Mediterranean diet and it decreases chronic inflammation by interfering with arachidonic acid and NF-κB signaling pathways. Specifically, the different components of EVO are able to confer advantages in terms of health in their site of action. For instance, oleic acid displays a protective effect in liver dysfunction and gut inflammation, whereas phenolic compounds protect colon cells against oxidative damage and improve the symptoms of chronic inflammation in IBD. Given the biological properties of EVO, we investigated whether its administration is able to confer protection in a mouse model of dextrane sodium sulfate (DSS)-induced colitis. Four EVO cultivars from the Apulian Region of Italy, namely Ogliarola (Cima di Bitonto), Coratina, Peranzana and Cima di Mola, respectively, were used. Administration of EVO resulted in reduced body weight loss in our colitis model. Furthermore, mice treated with Ogliarola, Coratina and Cima di Mola EVO displayed a reduction of rectal bleeding and IL-1β, TGFβ, IL-6 gene expression levels. Furthermore, Ogliarola, Coratina and Peranzana EVO administration ameliorated intestinal permeability and histopathological features of inflammation. Our data further validate the well-known positive effects of EVO supplementation in promoting human health and suggest the bona fide contribution of EVO in preventing onset and reducing progression of intestinal inflammation.

## 1. Introduction

Inflammatory bowel disease (IBD) is a chronic immune-mediated inflammatory disorder that affects the gastrointestinal tract [1]. The onset of IBD occurs in early adult life, although individuals of any age can be affected. Moreover, the majority of people who develop IBD may relapse, while a minority of them develop colorectal cancer (CRC) as a long-term complication [2].

The incidence of IBD has been rapidly increasing worldwide, with a peak in newly industrialized countries that adopt Western lifestyle. Although incidence is stabilizing in Western countries, the prevalence surpasses 0.3% [3]. There are two main types of IBD phenotypes, Crohn’s disease (CD) and ulcerative colitis (UC), each defined by specific clinical, pathological, endoscopic and radiological features [4].

Although there is no universal consensus due to the lack of rigorous evidence and the intrinsic heterogeneity of IBD, several diets such as the low-fermentable oligosaccharide, disaccharide, monosaccharide, and polyol diet (Low-FODMAP), the carbohydrates exclusion diet, the Mediterranean diet, the low-gluten diet and others, have shown anecdotal success in some patients [5]. To date, no endorsement of a specific nutritional regimen has been given however, following medical advice, personalized diets are considered adjuvant therapies when able to reduce the chronic inflammatory state of IBD patients, limiting the exacerbation and symptoms of the disease and preventing its progression to CRC.

Recent analyses underscore that the health benefits of olive oil could also depend on its phenolic constituents. Extra-virgin olive oil (EVO) is particularly enriched with these molecules (1%–2% of the total), that are completely absent in other type of oils derived from seeds or fruits [6]. Secoiridoids, such as oleuropein, ligstroside and oleocanthal, and their derivative phenolic alcohols, such as hydroxytyrosol (HT) and tyrosol, represent up to 90% of total phenolic compounds in EVO, with flavonoids and lignans covering the remaining 10% [7]. The concentration of phenolics in virgin olive oil depends on various agronomic (genetic and geographical origin of the fruits) and technological factors [8]. Monovarietal EVOs display diverse phenolic profiles, mainly due to different abundance of secoiridoids and their derivatives [9]. Importantly, the process utilized for the extraction of olive oil has a great influence on the phenol concentration: the manual crushing of the olives, typical of EVO, guarantees the preservation of these minor components, that are irreversibly lost with either chemical or physical procedures. Finally, the differences in phenolics abundance and composition might contribute to the health beneficial properties so that different monovarietal virgin olive oils would exert different effects at the cellular level [10], also due to their specific level of absorption and bioavailability. Presumably, the multiple phenolic compounds in virgin olive oil act in a synergistic or complementary way to confer benefits to the whole organism [11].

The antioxidant action of the phenolic compounds is more efficient in the gastrointestinal tract [11]. Indeed, the phenolic compounds in olive oil protects colon cells against injury induced by hydrogen peroxide [12]. Moreover, via direct interactions with cellular signaling pathways regulating cell growth, differentiation and metastasis in the gastrointestinal tract, phenolics can also exert a chemopreventive action [13].

Besides the above general evidence, several studies pointed to the contribution of single phenolic compounds to gut inflammation and related disorders.

Intriguingly, the variable concentration of secoiridoids and their derivatives in monovarietal virgin olive oil might differently impact on their health-promoting properties. Quintero-Flórez et al. demonstrated that five different Spanish olive oil cultivars differ in the bioaccessibility of phenolics, hence, in their different biological properties [10]. In addition, Yamada and colleagues highlighted that the cultivar-specific phenolic composition of various Tunisian olive oils associates with diverse inhibitory effects on inflammatory cytokines release [14]. Finally, in a comparison between two monovarietal olive oils, Incani et al. illustrated that the highest concentration of total phenols is more efficient in counteracting the pro-oxidant effect of dietary oxidized lipids in human intestinal cells [15]. Therefore, while the consumption of EVO per se is generally recommended, a meticulous characterization of monocultivar EVOs could be more informative for a more targeted nutraceutical choice.

In the present study, we analyzed whether multi-cultivar blend EVO from Apulian cultivars called “Ogliarola” (Cima di Bitonto), “Coratina”, “Peranzana “and “Cima di Mola” conferred protection against a dextrane sodium sulfate (DSS)-induced colitis mouse model.

## 2. Material and Methods

### 2.1. Mouse Model and Induction of Colitis

Eight-week-old male C57BL/6 mice were provided by Charles River Laboratories (Calco, Lecco, Italy). All mice were housed under pathogen-free conditions in a temperature-controlled room (23 °C) on a 12-h light/dark cycle and fed specific or control diets and water ad libitum. Animals were randomized based on body weight and were divided into different groups called by the names of the different olive oil monocultivars plus a control group: Ogliarola (Cima di Bitonto), Cima di Mola, Coratina, Peranzana and Placebo, the latter consisting of a physiological solution of sodium chloride 0.9% (*n* = 10 mice per group). EVOs were extracted from individual olive trees of four olive cultivars (Coratina, Cima di Mola, Ogliarola, Peranzana), originating from the provinces of Bari and Foggia (Apulia region, Southern Italy) and collected during two consecutive harvesting seasons (2013/14 and 2014/15). Colitis was induced by administration of 5% (*w*/*v*) dextran sodium sulfate (DSS; molecular mass 40 kDa; TDB consultancy, AB Uppsala, Sweden) in drinking water for 10 days. EVOs were administered daily by oral gavage starting a day prior to DSS administration throughout the whole duration of the experiment. Colitis symptoms were assessed daily. In particular, changes in body weight were recorded and stool consistency and rectal bleeding monitored daily. Hemoccult was scored as follows: 1, normal; 2, trace positive; 3, strong positive; and 4, gross bleeding. After the 10-days DSS treatment, all mice were sacrificed. The Ethical Committee of the University of Bari approved this experimental set-up, which also was certified by the Italian Ministry of Health in accordance with internationally accepted guidelines for animal care.

### 2.2. Histology

Colon specimens were snap-frozen or fixed in 10% formalin (24 h), dehydrated and embedded in paraffin. Distal colon sections (5 µm) were stained with hematosilin and eosin (H&E). Histopathological scoring was performed using an established semiquantitative score ranging from 0 to 6 based on infiltration of inflammatory cells and epithelial damage (1 = few inflammatory cells, no epithelial degeneration; 2 = mild inflammation, few signs of epithelial degeneration; 3 = moderate inflammation, few epithelial ulcerations; 4 = moderate to severe inflammation, ulcerations in 25%–50% of the tissue section; 5 = moderate to severe inflammation, large ulcerations in >50% of the tissue section; 6 = severe inflammation and ulcerations of >75% of the tissue section) [16].

### 2.3. In Vivo Intestinal Permeability Assay

Intestinal permeability was examined in mice in vivo on the day of sacrifice. Mice were gavaged with 0.6 mg/g body weight of fluorescein isothiocyanate (FITC)-conjugated dextran (Sigma, S Louis, MO, USA; molecular mass 3–5 kDa) for 4 h. Blood was collected, and FITC concentrations were measured in plasma (Victor Fluorimeter, PerkinElmer, MA, USA).

### 2.4. RNA Extraction and Real-Time Quantitative PCR

Total RNA was isolated by Qiazol reagent (Qiagen) following the manufacturer’s instructions. RNA was treated with DNase I (Ambion). RNA purity was checked by spectrophotometer and RNA integrity by examination on agarose gel electrophoresis. cDNA was synthesized retrotranscribing 4 μg of total RNA in a total volume of 100 μL using a High Capacity DNA Archive Kit (Applied Biosystems) following the manufacturer’s instructions.

Real-time quantitative PCR (RTqPCR) primers were designed using Primer Express software. PCR assays were performed in 96-well optical reaction plates using the QuantStudio5 machine (Thermo Fisher Scientific). PCR assays were conducted in triplicate wells for each sample. Baseline values of amplification plots were set automatically, and threshold values were kept constant to obtain normalized cycle times and linear regression data. The reaction mixture per well used were as follows: 10 AL Power Syber Green (Thermo Fisher Scientific), 2.4 AL of primers at the final concentration of 150 nmol/L, 4.6 AL RNase free water and 3 AL cDNA (60 ng). For all experiments, PCR conditions used were as follows: denaturation at 95 °C for 10 min, followed by 40 cycles at 95 °C for 15 s, then at 60 °C for 60 s. Quantitative normalization of cDNA in each sample was performed using GADPH as internal control. Relative quantification was performed using the ΔΔCT method. Validated primers for RTqPCR are available upon request.

### 2.5. Statistical Analysis

Results are expressed as mean ± SD as indicated in the figure legends. Statistical significance was determined by the paired Student t test or ANOVA analysis of variance (Kruskal–Wallis) with the Bonferroni post hoc test, as appropriate. All statistical calculations were performed with GraphPad 5.00 for Windows software (GraphPad Software, San Diego, CA, USA). Two-sided *p* values (*p* < 0.05, *p* < 0.01, *p* < 0.001) are considered significant. Grubbs’ test was used to determine whether one of the values was a significant outlier (GraphPad Software).

## 3. Results

### 3.1. Apulian EVO Reduce Inflammation in Mouse Model of Colitis

**Figure 1:**
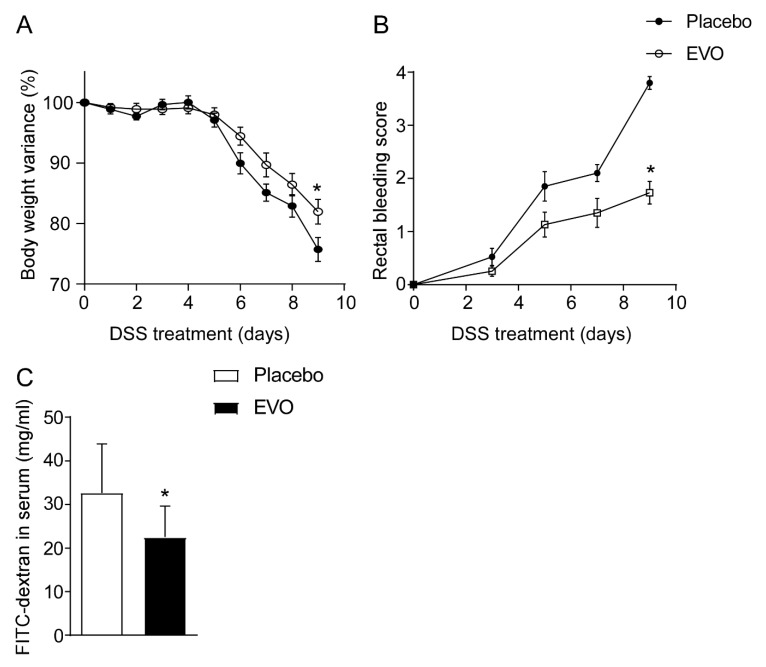
EVO administration confers protection against dextrane sodium sulfate (DSS)-induced colitis. (**A**) Percentage of body weight variance during DSS treatment, (**B**) visible rectal bleeding score, (**C**) in vivo intestinal permeability measurement after DSS-induced intestinal inflammation in C57Bl/J6 mice. All values represent means ± SD. Statistical significance comparing EVO versus Placebo (* *p* < 0.05) assessed by Mann–Whitney’s U test.

EVOs from Apulia represent almost 40% of the overall Italian production [17]. Apulian olive oils have been classified based on their chemical composition (e.g., free acidity, peroxide value, chlorophyll content, sterol, fatty acid and triacylglycerol) and on their nuclear magnetic resonance (NMR) profile [18]. Recently, Girelli et al. correlated NMR spectra of Apulian cultivars to their genetic profiles and pedo-climatic aspects observing a high variability in the phenolic pattern [19]. The protective effects of EVOs within the Mediterranean diet are well-recognized and they have been mostly attributed to the high content of MUFAs that could act in synergy with other minor components, such as oleuropein and hydroxytyrosol. In this context, we first investigated whether a multi-cultivar blend of EVO conferred protection against a DSS-induced colitis mouse model. EVO treatment significantly reduced the typical symptoms of intestinal inflammation including body weight loss and rectal bleeding (Figure 1A,B). In line with these data, EVO administration improved in vivo intestinal permeability compared to placebo (Figure 1C).

### 3.2. EVOs from Apulian Cultivars “Ogliarola”, “Coratina”, “Peranzana” and “Cima di Mola” Reduce Intestinal Inflammation in DSS-Treated Mice

**Figure 2:**
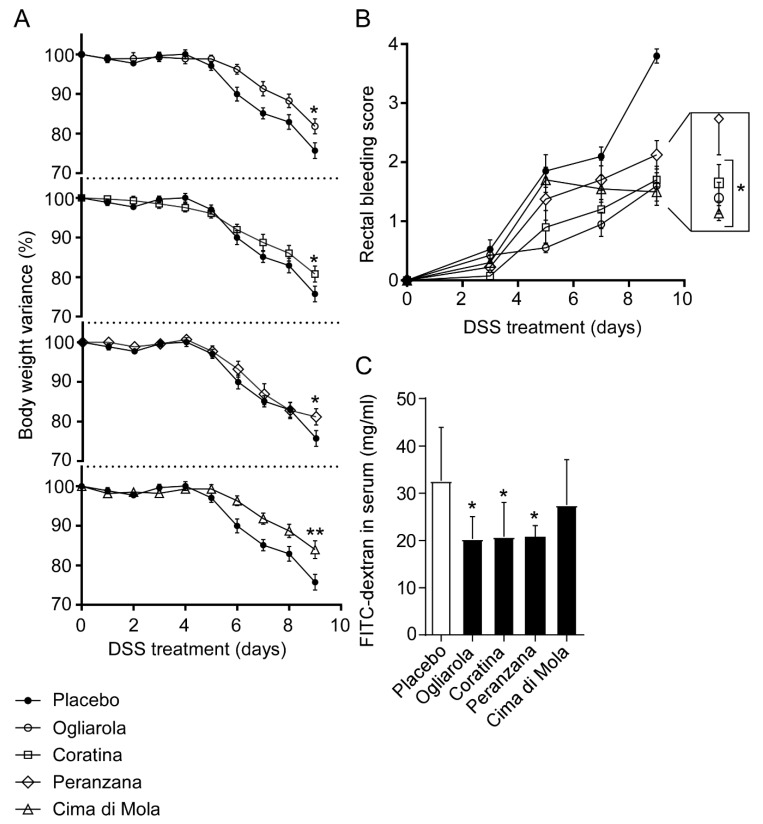
Apulian EVO cultivars treatment reduces (**A**) body weight loss, (**B**) rectal bleeding and (**C**) intestinal permeability in DSS-treated mice. Mice were treated with Ogliarola, Cima di Mola, Coratina, Peranzana EVO and placebo by oral gavage starting a day prior to DSS administration through 10th day. (**A**) Percentage of body weight variance during DSS treatment, (**B**) visible rectal bleeding score, (**C**) In vivo intestinal permeability measurement after DSS-induced intestinal inflammation in C57Bl/J6 mice. All values represent means ± SD. Differences between means were tested for significance using 1-way ANOVA followed by the Bonferroni test (* *p* < 0.05).

We extended our study analyzing the anti-inflammatory potential of four monocultivars of EVO, namely “Ogliarola”, “Coratina”, “Peranzana” and “Cima di Mola”. The four Apulian monocultivars have been previously characterized through NMR fingerprints, each revealing specific levels of saturated and unsaturated fatty acids [20]. In particular, Coratina EVO presented the highest content of oleic and eicosenoic acids and the lowest content of the remaining fatty acids and total tocopherols compared to the other Apulian EVO cultivars. Furthermore, three-dimensional principal component analysis (PCA) revealed a degree of separation for Coratina EVO and a degree of overlap for Cima Di Mola, Ogliarola and Peranzana cultivars [20]. All the four cultivars significantly reduced body weight loss as compared to placebo (Figure 2A). Moreover, administration of Ogliarola, Coratina and Cima di Mola EVOs reduced rectal bleeding compared to mock-treated controls (Figure 2B). Furthermore, in Ogliarola-, Coratina- and Peranzana-treated mice, we observed a reduction of intestinal permeability compared to the placebo group (Figure 2C).

### 3.3. EVOs from Apulian Cultivars “Ogliarola”, “Coratina”, “Peranzana” and “Cima di Mola” Improve Intestinal Morphology and Down-Regulate Pro-Inflammatory Cytokines Levels

**Figure 3:**
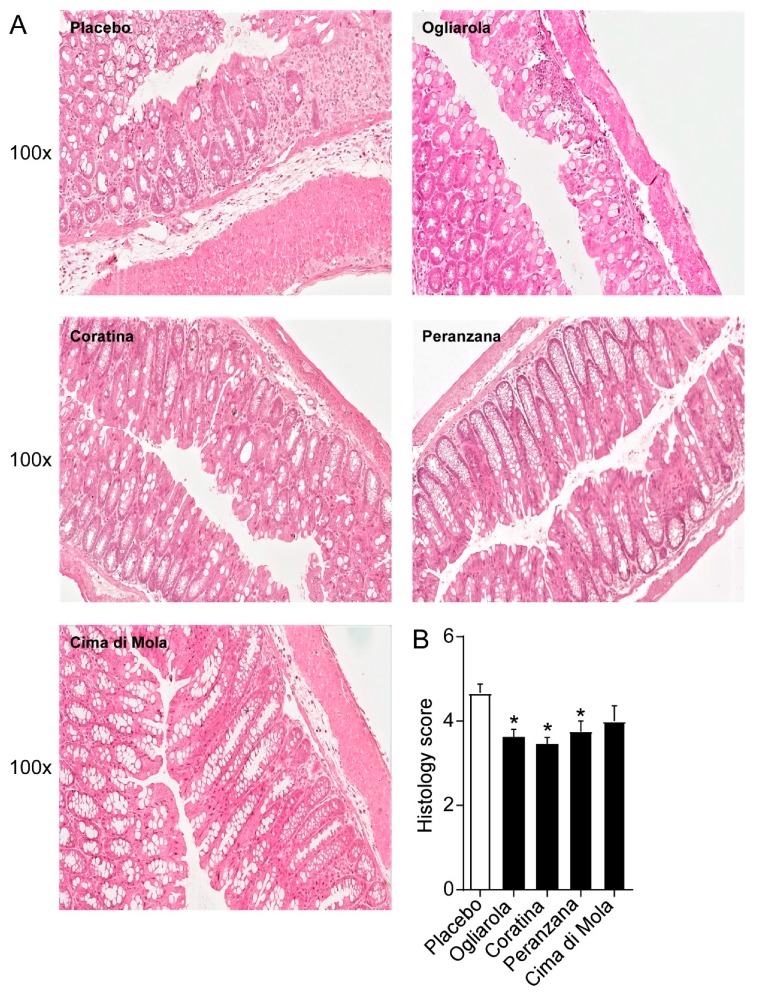
Apulian EVO cultivars treatment improves intestinal morphology in DSS-treated mice. (**A**) Representative H&E-stained colonic sections for Ogliarola, Cima di Mola, Coratina, Peranzana EVOO and placebo treated mice. (100× Magnification) (**B**) Histology score. All values represent means ± SD. Differences between means were tested for significance using 1-way ANOVA followed by the Bonferroni test (* *p* < 0.05).

As shown in Figure 3A, in mice treated with the placebo, DSS treatment induced the development of dystrophic goblet cells, crypt abscesses and an important neutrophil invasion as well as a thickening of submucosa and muscularis layers. Treatments with EVO cultivars drastically decreased neutrophil infiltration, number of dystrophic goblet cells and the presence of crypt abscess. Specifically, mice treated with Ogliarola, Coratina and Peranzana cultivars displayed a reduction of inflammatory infiltrate and epithelial damage compared to controls. A histopathological score method was applied and quantification of a histological disease index is shown (Figure 3B). These results point out that Ogliarola, Coratina and Peranzana cultivars, once in contact with the intestinal mucosa, carry on a remarkable anti-inflammatory protection.

**Figure 4:**
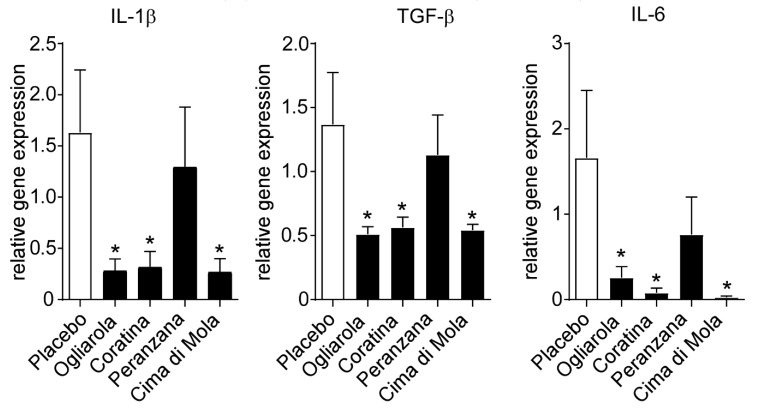
Apulian EVO cultivars treatment improves liver function and reduces inflammatory cytokines gene expression levels in DSS-treated mice. Gene expression analysis of colon is reported. GADPH was used as a housekeeping gene to normalize data and wild type mice as calibrators. The results are expressed as mean ± SEM. Differences between means were tested for significance using 1-way ANOVA followed by the Bonferroni test (* *p* < 0.05).

In order to explain the anti-inflammatory mechanisms of Apulian EVO cultivars against DSS-induced colitis, we studied inflammatory cytokine gene expression levels in colon samples. We found a significant downregulation of Il-1β, TGF-β and Il-6 gene expression levels in Ogliarola, Coratina and Cima di Mola-treated mice compared to placebo group (Figure 4). These data indicate that Ogliarola, Coratina and Cima di Mola cultivars confer protection from DSS-induced colitis through the reduction of inflammatory cytokine levels and inflammatory infiltrate (Figure 3).

## 4. Discussion

In the present work, we evaluated the effects of four Apulian EVO cultivars, namely Ogliarola (Cima di Bitonto), Coratina, Peranzana and Cima di Mola in a mouse model of DSS-induced colitis. Administration of EVO reduced body weight loss and rectal bleeding and improved intestinal permeability. In particular, mice treated with Ogliarola, Coratina and Cima di Mola EVO displayed a reduction of rectal bleeding and pro-inflammatory cytokines expression levels, while Ogliarola, Coratina and Peranzana EVO administration ameliorated intestinal permeability and histopathological features of inflammation, demonstrating positive effects of EVO supplementation in the prevention and development of colitis.

The gastrointestinal tract represents the interface between ingested food and the rest of the body. Therefore, the maintenance of gut homeostasis is mandatory to guarantee the healthy status of the whole-body. Numerous observational studies have identified dietary patterns contributing to the risk of IBD. An inverse association between a diet rich in fibers, fruit and vegetables and a decrease risk of IBD has been demonstrated. On the contrary, individuals consuming great amounts of meat and fats, particularly polyunsaturated fatty acids (PUFAs) and omega-6 (n-6) fatty acids, have shown a higher risk of IBD development [21]. The European Prospective Investigation into Cancer (EPIC) study revealed that high consumption of sugar and soft drinks, together with low ingestion of vegetables correlates with the onset of UC [22]. The same study also pinpointed to an association between increased dietary intake of linoleic acid and higher incidence of UC [19]. Linoleic acid is an essential n-6 PUFA that is converted to arachidonic acid, the metabolic precursor of pro-inflammatory eicosanoids. In contrast, people who consume higher levels of docosahexanoic acid, an omega-3 (n-3) PUFA, are less frequently diagnosed with UC [23,24]. Another prospective study, the Nurses’ Health Study, has reported that a greater consumption of long-chain n-3 PUFAs and a higher ratio of n-3:n-6 PUFAs is protective for the development of UC, while large consumption of fibers, particularly from fruits, is associated with 40% reduction in risk of CD [25]. Moreover, several studies have highlighted that high fat dietary intake (mainly saturated fatty acids) can contribute to the development of intestinal inflammation and CRC [24,26]. Conversely, the consumption of olive oil is associated with a decreased incidence of CRC in humans and suppression of inflammation in a rat model of IBD [27]. In this context, a growing body of evidence suggests that the Mediterranean diet, having olive oil as the main source of fat, protects against the development and progression of a multitude of diseases, including obesity, metabolic syndrome, cardiovascular diseases and cancer [28].

In particular, EVO decreases chronic inflammation by interfering with the arachidonic acid and NF-κB pathways, largely involved in the promotion of the inflammatory response. Specifically, arachidonic acid represents the starting point of the inflammatory response. It is processed by cyclooxygenase (COX) to form prostaglandin, a precursor of prostanoids and thromboxane, which then act in autocrine and paracrine manners to maintain a proinflammatory microenvironment. Concomitantly, pro-inflammatory cytokines induce the expression of NF-κB that, in turn, amplifies the inflammatory response by regulating its target genes. Intriguingly, a Mediterranean diet enriched with EVO decreases serum concentration of COX and related inflammatory cytokines [29], and attenuates the NF-κB pathway, thus lowering inflammation [30].

Usually, the beneficial effects of olive oil have been ascribed to the high content of monounsaturated fatty acids (MUFA). The principal MUFA in olive oil is oleic acid (18:1 n-9), which account for up to 83% of the total lipid composition. Oleic acid plays a protective effect against the onset of several diseases, including liver dysfunction and gut inflammation [31]. Indeed, the supplementation of oleic acid in mice presenting with increased expression of inflammatory markers and crypt proliferative genes has been proven to be beneficial by decreasing intestinal inflammation and CRC progression [32]. Moreover, a lower level of mucosal oleic acid has been observed in human with IBD and in rat treated with trinitrobenzene sulfonic acid (TNB)-induced colitis [33].

Phenolic compounds can also modulate the inflammatory response, thus contributing to improve symptoms of chronic inflammation in IBD and other immuno-mediated inflammatory diseases, and reduce the risk of chronic inflammation pathologies development [34]. Notably, an EVO diet supplemented with EVO-extracted polyphenols has more pronounced protective effects than an EVO diet alone in a murine DSS-induced chronic colitis model, as shown by the downregulation of inflammatory genes, such as tumor necrosis factor α (TNFα) and monocyte chemoattractant protein 1 (MCP1) [35].

Oleuropein, responsible for the bitter taste of the oil, is a bioactive secoiridoid particularly abundant in green olive drupes [36]. Oleuropein is able to modulate senescence-associated inflammatory phenotype, via the downregulation of senescence/inflammation markers such as interleukin-6 (IL-6), metalloprotease secretion and COX-2 [37]. Ex vivo treatment with oleuropein of colonic biopsy from patients with colitis, decreases the expression of IL-17 and COX-2, thus resulting in the amelioration of the inflammatory process [38]. Moreover, in mouse models of colitis and colitis-associated CRC, oleuropein exerts a protective effect on acute inflammatory relapse and transition to chronic inflammation, thereby limiting the activation of main transcription factors involved in cancer cells proliferation [39].

Hydroxytyrosol, an oleuropein derivative which originates during the maturation of olives and storage of oil, has anti-inflammatory and anti-aggregant effects that widely contribute to its cardiovascular protective action [40]. Intriguingly, hydroxytyrosol decreases the acrylamide-induced oxidative stress in a colon cancer cell line by acting on ROS scavenging systems [41]. Moreover, an EVO enriched-diet with hydroxytyrosol displays a protective effect in a DSS-induced murine chronic colitis model [42]. Notably, different from other phenolic compounds, hydroxytyrosol is readily bioavailable, so it could rapidly exert its function in target tissues [43].

Oleocanthal is abundant in newly pressed EVO, and its pungency induces a strong stinging sensation in the throat, to the point that throat irritation intensity positively correlates with oleocanthal concentration [44]. Oleocanthal boasts an anti-inflammatory action similar to the non-steroidal anti-inflammatory drug ibuprofen. Despite a dissimilar molecular structure, both oleocanthal and ibuprofen inhibit the same COX enzymes in the prostaglandin-biosynthesis pathway [45]. In particular, oleocanthal hampers COX function by blocking p38/CREB phosphorylation, involved in the regulation of COX transcription [46]. Given the protective effects displayed by non-steroidal anti-inflammatory drugs in IBD and CRC, it is possible that also oleocanthal administration would result in amelioration of disease conditions [47].

In this scenario, we observed a reduction of intestinal inflammation in a DSS-induced colitis mouse model and the peculiar effects observed in the four Apulian EVO cultivars could be ascribed to their variability in the phenolic pattern.

Altogether, this evidence suggests that the nutraceutical properties of EVO phenolic compounds are strongly associated with an anti-inflammatory profile, thus indicating that a wide range of chronic inflammatory diseases, including those of the gastrointestinal tract, could benefit from these biologically active compounds.

## 5. Conclusions

The importance of diet in the management of IBD has become increasingly recognized and there is an increasing effort in studying the effect of nutrients that could modulate the intestinal inflammatory state and promote gut health. Specific IBD diets may be helpful to defined groups of patients, however, given this disease’s heterogeneity and the risk of weight loss and malnutrition, there is no one-size-fits-all diet for these patients. Supplementation with unsaturated fatty acids to decrease inflammation in gastrointestinal diseases has been based on epidemiologic observations, mainly in IBD [48]. Dietary nutrients are the most common luminal antigens in the bowel and may influence intestinal inflammation. Hence, several dietary habits and molecular mechanisms could modulate inflammatory mediators, modify microbiota composition and affect gut permeability. While high intake of total fat and omega-6 fatty acids has been associated with increased risk of developing IBD, high vegetable and fruit intake decreases its risk [21]. Interestingly, IBD incidence is significantly lower in Mediterranean countries [49] where EVO consumption is elevated. Our data suggest the importance of EVO monovarietal cultivars composition and their specific impact on intestinal inflammation in a mouse model of colitis. For this reason, the use of Apulian EVO cultivars enriched in minor components could be an adjuvant nutritional-therapeutic option to decrease the inflammatory symptoms when colitis is diagnosed.
